# TLR4-dependent effects of ISAg treatment on conventional T cell polarization *in vivo*

**DOI:** 10.1080/19768354.2019.1610059

**Published:** 2019-04-25

**Authors:** Sung Won Lee, Hyun Jung Park, Seo Hyun Kim, Sooyong Shin, Kyung Hee Kim, Sang Jae Park, Seokmann Hong, Sung Ho Jeon

**Affiliations:** a Department of Integrative Bioscience and Biotechnology, Institute of Anticancer Medicine Development, Sejong University, Seoul, Korea; b Department of Life Science and Multidisciplinary Genome Institute, Hallym University, Chuncheon, Korea; c Medience Co., Ltd., Chuncheon Bioindustry Foundation, Chuncheon, Korea

**Keywords:** *Angelica gigas*, TLR4, Th1 polarization, Treg cells

## Abstract

We recently demonstrated that the polysaccharide component of the Korean medicinal herb *Angelica gigas* (immuno-stimulatory fraction of *A. gigas*; ISAg) induces anticancer effects in mice by activating natural killer (NK) and natural killer T (NKT) cells. However, it is unclear whether the use of ISAg *in vivo* can affect the differentiation of conventional T cells. Here, we investigated the effects of ISAg on the activation of conventional CD4^+^ and CD8^+^ T cells. We found that the administration of ISAg induced the polarization of CD4^+^ T cells toward the acquisition of the Th1 phenotype *in vivo*. Additionally, in mice treated with ISAg, CD8^+^ T cells produced more IFNγ than in control mice treated with PBS. Moreover, treatment with ISAg activated CD4^+^ and CD8^+^ T cells as well as NK and NKT cells, resulting in the secretion of Th1-type cytokines in a toll-like receptor 4 (TLR4)-dependent manner, implying that TLR4 is critical for an optimal Th1 response. Interestingly, ISAg treatment increased the number of Foxp3^+^ Treg cells, but not of Th2 cells, compared to control mice treated with PBS, indicating that ISAg possesses an immunomodulatory capacity that can control adaptive immune responses. Taken together, our results indicate that ISAg possesses a Th1-enhancing activity that could be used to treat Th2-mediated allergic immune diseases such as atopic dermatitis.

## 1. Introduction

*Angelica gigas* Nakai (i.e. Korean angelica or Dang Gui) is used as a traditional herbal medicine in East Asian countries. Decursin, decursinol angelate, and pectic polysaccharide (angelan) isolated from *A. gigas* extracts elicit potent anticancer effects, such as the direct inhibition of tumor cell proliferation, adhesion, and invasion (Han et al. 2006; Lee et al. 2009; Kim et al. 2018a). However, these components can exert different immune responses (suppression and activation of the immune system). Decursin and decursinol angelate from *A. gigas* extracts were found to enhance the anti-inflammatory activity of macrophages via heme oxygenase-1 expression (Cho et al. 2015) but suppress the production of interleukin (IL) 6 and TNFα in mice treated with dextran sulfate sodium (Oh et al. 2017). However, angelan from *A. gigas* extracts was discovered to induce the rapid secretion of IL6 and interferon γ (IFNγ) by splenocytes (Han et al. 1998) and directly enhance the immune activation of B cells and macrophages (Han et al. 2006). Angelan was also found to improve the activation and maturation of dendritic cells (DCs) via the toll-like receptor 4 (TLR4) signaling pathway (Kim et al. 2007). These previous studies suggest that specific components from *A. gigas* extracts can elicit different immune responses.

TLRs regulate the development of adaptive immune responses that are mediated by T and B cells via complex immune system interactions (Manicassamy and Pulendran 2009). Of note, in TLR4-mediated activation of the immune system, antigen-presenting cells (APCs) such as DCs and macrophages are the major producers of IL12, whereas natural killer (NK) and natural killer T (NKT) cells are responsible for producing IFNγ (Lee et al. 2013, 2018a, 2018c; Manicassamy and Pulendran 2009). Such an increased production of IL12 by antigen-presenting cells after stimulation by NK and NKT cell-derived IFNγ is essential for optimal Th1 cell development (Fujii et al. 2007; Manicassamy and Pulendran 2009; Lee et al. 2018b).

Recently, we demonstrated that the immuno-stimulatory fraction of *A. gigas* (ISAg), which is composed of water-soluble polysaccharides, elicits anticancer effects via the activation of NK and NKT cells, which are known to play important roles in innate immune responses (Kim et al. 2018b). Thus, in this study, we investigated whether ISAg influences the activation and polarization of conventional T cells such as CD4^+^ and CD8^+^ T cells *in vivo*. We found that orally administered ISAg skewed these T cells toward the adoption of a Th1 phenotype, resulting in the production of IFNγ and TNFα. This response was also dependent on the TLR4 signaling pathway. Furthermore, orally administered ISAg resulted in an increase in the frequency of Treg cells, but not of Th2 cells. Our results suggest that ISAg could be used as a therapeutic adjuvant to treat allergic diseases such as asthma and atopic dermatitis (AD), which are known to be caused by a Th1/Th2 imbalance.

## 2. Materials and methods

### 2.1. Mice and reagents

Wild-type (WT) C57BL/6 (B6), C3H/HeN (TLR4-WT), and C3H/HeJ (TLR4-mutant) mice were obtained from Jung Ang Lab Animal Inc. (Seoul, Korea). The IL12p40 reporter (Yet40) was provided by Dr. R. Locksley (University of California at San Francisco, CA, USA). All mice used in this study were maintained at either Hallym University or Sejong University. The animal experiments were approved by the Institutional Animal Care and Use Committee at both Hallym University (Hallym 2016-34) and Sejong University (SJ-20160705). All experiments were blinded and randomized using age- and sex-matched mice, which were euthanized by CO_2_ asphyxiation prior to the experiments. ISAg was isolated from *A. gigas* Nakai roots, as described previously (Kim et al. 2018b). LPS was obtained from Sigma-Aldrich (St. Louis, MO, USA).

### 2.2. Flow cytometry

The following monoclonal antibodies (mAbs) were obtained from BD Biosciences (San Jose, CA, USA): phycoerythrin (PE)- or allophycocyanin (APC)-conjugated anti-NK1.1 (clone PK-136); PE-Cy7-conjugated anti-CD11b (clone M1/70); PE-conjugated anti-IL12p40 (clone C15.6); biotin-conjugated CD86 (clone GL1); PE-Cy7- or APC-conjugated anti-CD3ϵ (clone 145-2C11); PE-Cy7-, or APC-conjugated anti-CD4 (clone RM4-5); PE-Cy7-, or APC-conjugated anti-CD8α (clone 53-6.7); APC-conjugated anti-CD25 (clone PC61); PE-Cy7- or APC-conjugated anti-CD11c (clone HL3); PE-conjugated anti-TNFα (clone XP6-XT22); and PE-conjugated anti-IgG1 (κ isotype control). The following mAbs from Thermo Fisher Scientific were used: APC-conjugated anti-F4/80 (clone BM8); PE-conjugated anti-IL4 (clone BVD6-24G2); PE-conjugated anti-Foxp3 (clone XMG1.2); and PE-conjugated anti-IFNγ (clone XMG1.2). Flow cytometry data were acquired using a FACSCalibur instrument (Becton Dickinson Inc., San Jose, CA, USA) and analyzed using the FlowJo analysis tool (Tree Star Inc., Ashland, OR, USA). For surface antibody staining, cells were collected and washed twice with FACS buffer (PBS containing 0.5% bovine serum albumin). The cells were preincubated with purified anti-CD16/CD32 mAbs (BD Bioscience, Bedford, MA, USA) on ice for 10 min to block nonspecific binding to Fc receptors and were then stained with fluorescent mAbs.

### 2.3. Intracellular cytokine staining

For intracellular staining, splenocytes were incubated with brefeldin A in RPMI 1640 medium (10 μg/mL) at 37°C for 2 h. Cells were stained for specific surface markers and fixed with 1% paraformaldehyde. After permeabilization with 0.5% saponin (Sigma-Aldrich), cells were stained with the indicated mAbs (PE-conjugated anti-IFNγ, anti-TNFα, anti-IL12p40, anti-IL4, and PE-conjugated isotype control rat IgG) for 30 min. Fixation and permeabilization were performed using a Foxp3 staining kit (eBioscience) with the indicated mAbs (PE-conjugated anti-Foxp3 and PE-conjugated isotype control rat IgG). More than 5000 events per sample were acquired using the FACSCalibur instrument.

### 2.4. Cell culture

A single-cell suspension of splenocytes was prepared in RPMI 1640 complete medium (Gibco BRL, USA) supplemented with 10% FBS, 10 mM HEPES, 2 mM L-glutamine, 100 units/mL penicillin–streptomycin, and 5 mM 2-mercaptoethanol.

### 2.5. *In vivo* ISAg administration

To evaluate the dose-dependent effects of ISAg on innate immune responses, Yet40 B6 mice were given ISAg via oral gavage at a dose of 4 mg/mouse, three times per week for 4 weeks. For experiments to test the involvement of TLR4 signaling in ISAg-mediated innate immune responses, C3H/HeN and C3H/HeJ mice were orally administered 4 mg ISAg three times per week for 4 weeks. For all of these experiments, the positive control LPS (2 μg/mouse) was injected intraperitoneally (i.p.) once per week for 4 weeks.

### 2.6. Statistical analysis

Statistical significance was analyzed using the Excel statistical analysis tool (Microsoft, Redmond, WA, USA). The comparison of two groups was performed by the Student's *t* test. Values of *P less than 0.05 and **P less than 0.01 were considered statistically significant in the Student's *t* test. VassarStats statistical software (http://vassarstats.net/anova2u.html) was used for the two-way analysis of variance (ANOVA). Values of ^#^*P* less than 0.05 and ^##^*P* less than 0.01 were considered statistically significant in the two-way ANOVA.

## 3. Results and discussion

### 3.1. Oral administration of ISAg induces IL12 production and maturation of antigen-presenting cells

**Figure 1.**
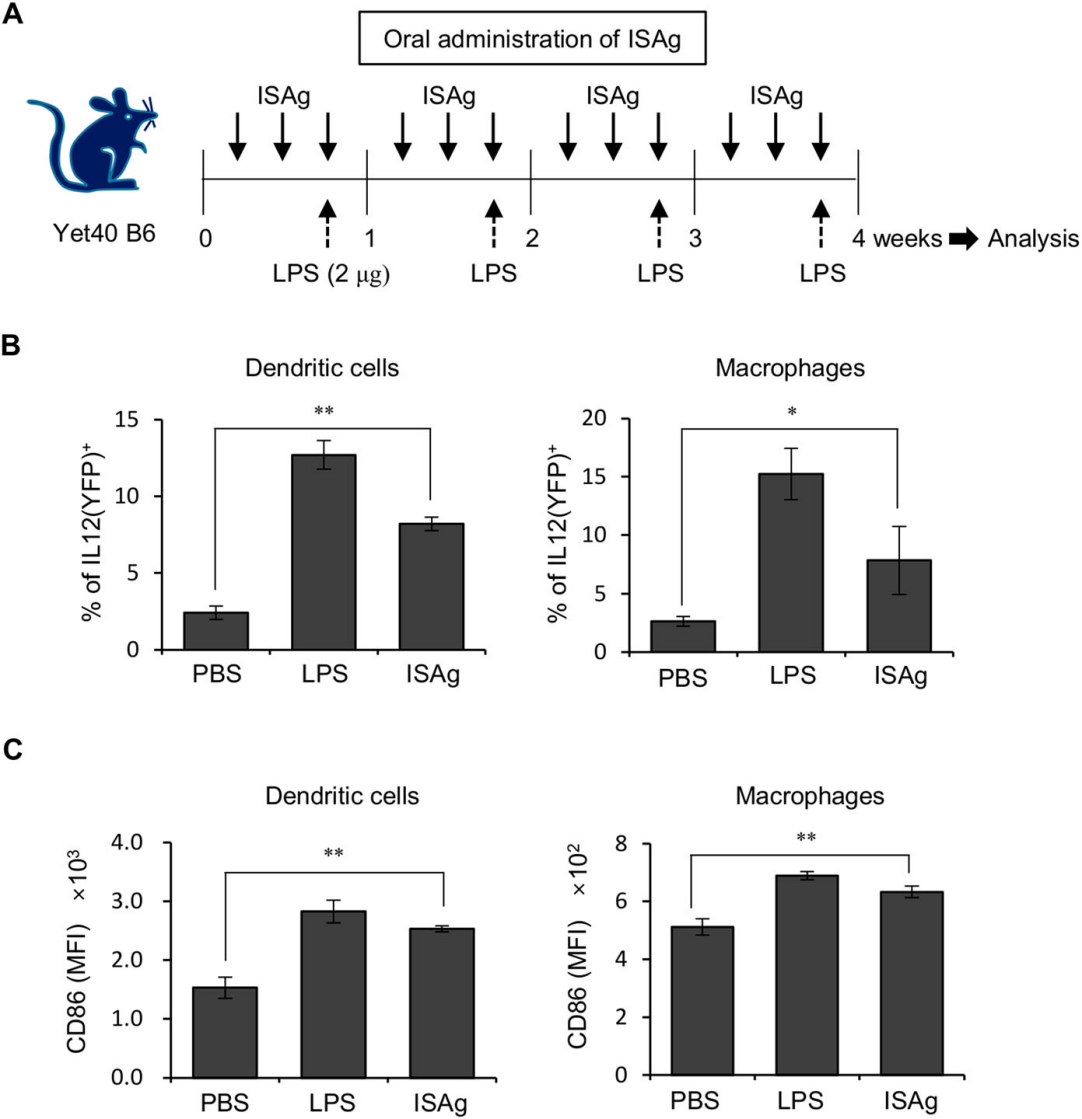
Oral administration of ISAg induces IL12 production and maturation of antigen-presenting cells. (A) Yet40 B6 mice were given 4 mg ISAg or PBS by oral gavage three times per week for 4 weeks. Positive control mice were injected (i.p.) with 2 μg LPS once per week for 4 weeks. Four weeks later, the levels of IL12p40 (YFP) (B) and CD86 (C) in splenic DCs (CD11c^+^) and macrophages (CD11c^−^CD11b^+^F4/80^+^) were measured by flow cytometry. The results are presented as the mean ± standard deviation (*n* = 4 per group; Student's *t* test; *P < 0.05, **P < 0.01).

Recently, we demonstrated that the water-soluble polysaccharide fraction of *A. gigas*, ISAg, elicits anticancer effects through cytotoxic activity of NK and NKT cells (Kim et al. 2018b). Since it is well established that the overproduction of IL12p40 by DCs leads to Th1 polarization of conventional CD4^+^ and CD8^+^ T cells (Mayordomo et al. 2018), we examined whether repeated oral administration of ISAg could induce IL12 production and the maturation of APCs such as DCs and macrophages. For this purpose, we used Yet40 (IL12p40 reporter) mice (Reinhardt et al. 2006) (Figure 1A) and found that orally administered ISAg could stimulate APCs, resulting in an increase in IL12 production (Figure 1B) and CD86 expression (Figure 1C). Moreover, ISAg treatment increased the number of total splenocytes as well as the frequency of inflammatory cytokine-producing immune cells (Data not shown). These findings indicate that ISAg-stimulated APCs possess the capacity to differentiate naive CD4^+^ T cells into Th1 cells.

### 3.2. TLR4 signaling is critical to ISAg-triggered cytokine production by NK and NKT cells

**Figure 2.**
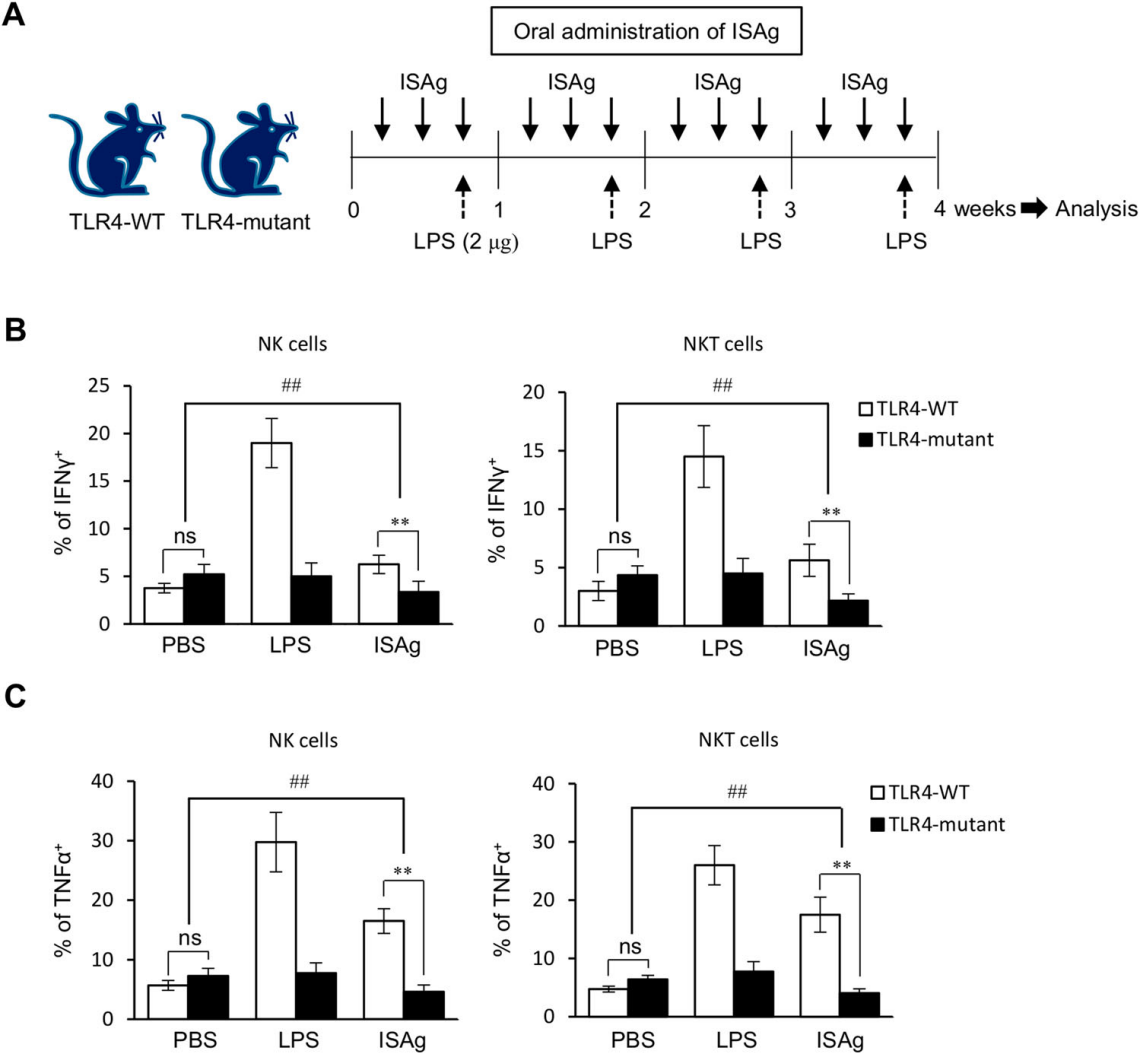
The TLR4 signaling pathway is critical for ISAg-stimulated cytokine production by NK and NKT cells. (A) C3H/HeN and C3H/HeJ mice were given 4 mg ISAg or PBS by oral gavage three times per week for 4 weeks. Positive control mice were injected (i.p.) with 2 μg LPS once per week for 4 weeks. Four weeks later, the levels of IFNγ (B) and TNFα (C) in NK and NKT cells were measured by flow cytometry. The results are presented as the mean ± standard deviation [*n* = 4 per group; Student's *t* test; **P < 0.01; two-way ANOVA (TLR4 mutant × ISAg treatment); ^##^*P* < 0.01].

We previously showed that ISAg-mediated IL12 production by DCs and macrophages is dependent on the TLR4 pathway (Kim et al. 2018b). However, since it was reported that TLR2 and TLR4 ligands directly activate NK and NKT cells (Martinez et al. 2010; Kim et al. 2012), we examined whether the activation of NK and NKT cells by ISAg could be regulated by TLR4 signaling. We administered ISAg orally to either TLR4-WT C3H/HeN or TLR4-mutant C3H/HeJ mice (Figure 2A) and measured the IFNγ and TNFα levels in NK and NKT cells by flow cytometry. As expected, the production of IFNγ and TNFα by NK and NKT cells was noticeably lower in ISAg-treated C3H/HeJ mice than in C3H/HeN mice (Figure 2B and C). These results indicate that NK and NKT cell activation by ISAg is TLR4 dependent. Based on our previous study that ISAg-triggered NK and NKT activation is mediated by IL12, it is more likely that ISAg indirectly activate NK and NKT cells via IL12 from APCs. Since it was reported that IFNγ can directly suppress the activation of type 2 innate lymphoid cells and basophils, which are critical for the induction of Th2 polarization (Molofsky et al. 2015; Park et al. 2016), the early production of IFNγ by NK and NKT cells after stimulation might antagonize Th2 polarization.

### 3.3. ISAg treatment induces the polarization of CD4^+^ T cells toward the acquisition of the Th1 phenotype and activation of CD8^+^ T cells via the TLR4 signaling pathway

**Figure 3.**
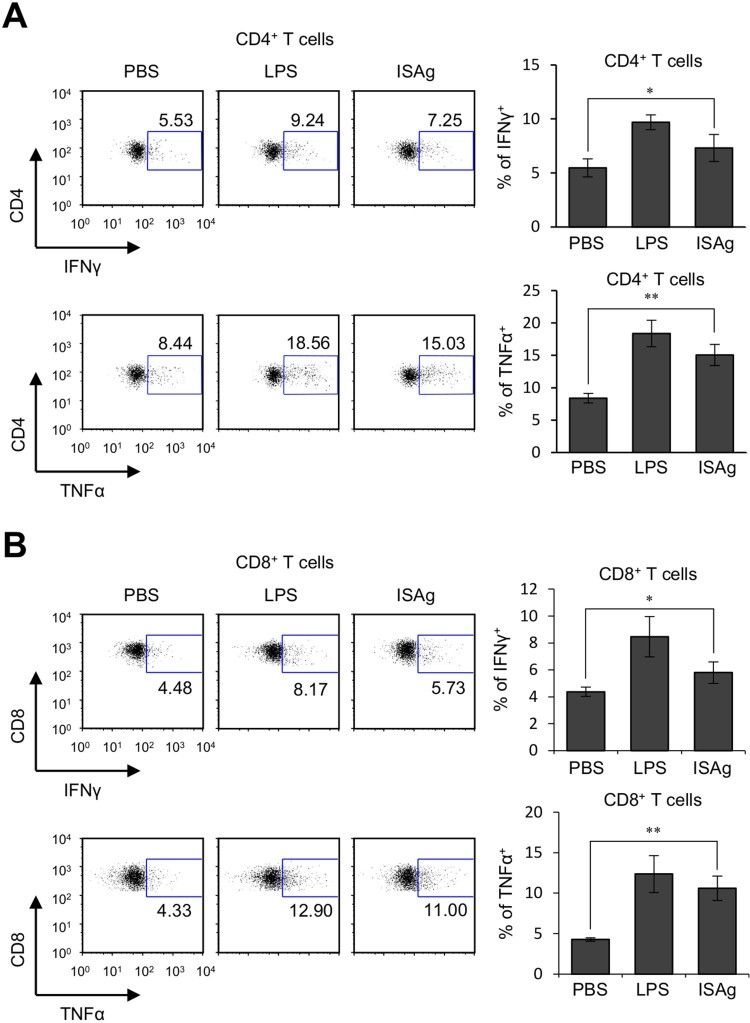
Orally administered ISAg promotes the polarization of CD4^+^ and CD8^+^ T cells toward adopting a Th1 phenotype. (A and B) Yet40 B6 mice were given 4 mg ISAg or PBS by oral gavage three times per week for 4 weeks. Positive control mice were injected (i.p.) with 2 μg LPS once per week for 4 weeks. Four weeks later, the levels of IFNγ and TNFα in CD4^+^ T cells (A) and CD8^+^ T cells (B) were measured by flow cytometry. The results are presented as the mean ± standard deviation (*n* = 4 per group; Student's *t* test; *P < 0.05, **P < 0.01).

Since our results showed that IL12 and IFNγ, which were increased in ISAg-treated mice, are essential for the Th1-type polarization of CD4^+^ T cells (Fujii et al. 2007; Zhang et al. 2008; Lee et al. 2013), we examined whether ISAg treatment could affect the differentiation of CD4^+^ T cells *in vivo*. To address this issue, we analyzed the cytokine profiles of CD4^+^ T cells after ISAg stimulation *in vivo*. We found that ISAg-treated CD4^+^ T cells produced high levels of IFNγ and TNFα, indicating that these cells became polarized toward adopting the Th1 phenotype (Figure 3A). Since inflammatory cytokines such as IL12 and IFNγ are known to help activate CD8^+^ T cells (Henry et al. 2008), we next examined whether ISAg treatment could affect the production of cytokines by CD8^+^ T cells. We found that ISAg treatment induced CD8^+^ T cells to produce IFNγ and TNFα, indicating that ISAg activates CD8^+^ T cells (Figure 3B).

**Figure 4.**
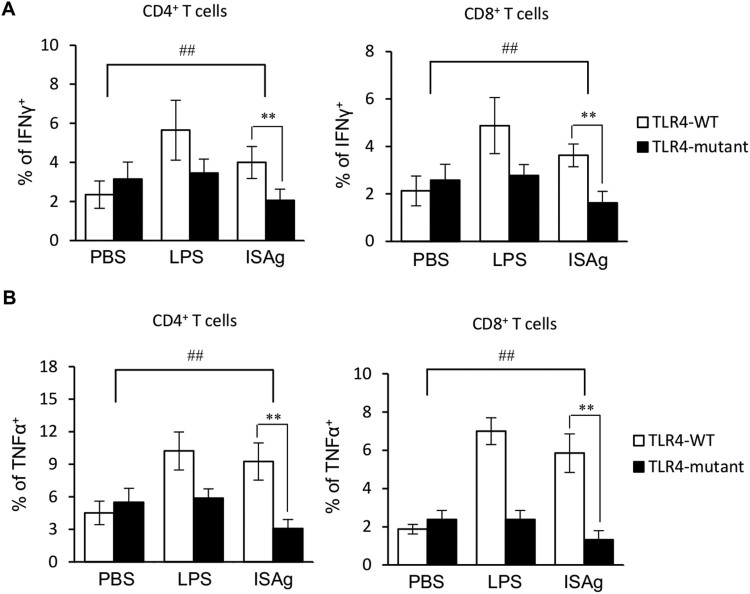
The TLR4 signaling pathway is critical for the ISAg-induced polarization of CD4^+^ and CD8^+^ T cells toward adopting a Th1 phenotype. (A and B) C3H/HeN and C3H/HeJ mice were given 4 mg ISAg or PBS by oral gavage three times per week for 4 weeks. Positive control mice were injected (i.p.) with 2 μg LPS once per week for 4 weeks. Four weeks later, the levels of IFNγ (A) and TNFα (B) in CD4^+^ and CD8^+^ T cells were measured by flow cytometry. The results are presented as the mean ± standard deviation [*n* = 4 per group; Student's *t* test; **P < 0.01; two-way ANOVA (genotype × treatment); ^##^*P* < 0.01].

Since ISAg can activate NK and NKT cells via the TLR4 signaling pathway like LPS, we examined whether the effects of ISAg on helper T cell differentiation could also be mediated by the TLR4 signaling pathway. To test this, ISAg was administered orally to either TLR4-WT or TLR4-mutant mice. We found that both Th1 differentiation and CD8^+^ T cell activation were mediated in a TLR4-dependent manner after ISAg treatment (Figure 4A and B). Collectively, these data demonstrate that the TLR4 signaling pathway plays an essential role in linking the innate and adaptive immune responses after ISAg treatment. Moreover, Consistent with our observations of ISAg-mediated NK and NKT activation, it is more likely that TLR4-dependent modulating effects of ISAg on CD4^+^ or CD8^+^ T cells are mediated via IL12 from APCs.

### 3.4. Oral administration of ISAg induces Treg expansion *in vivo*

**Figure 5.**
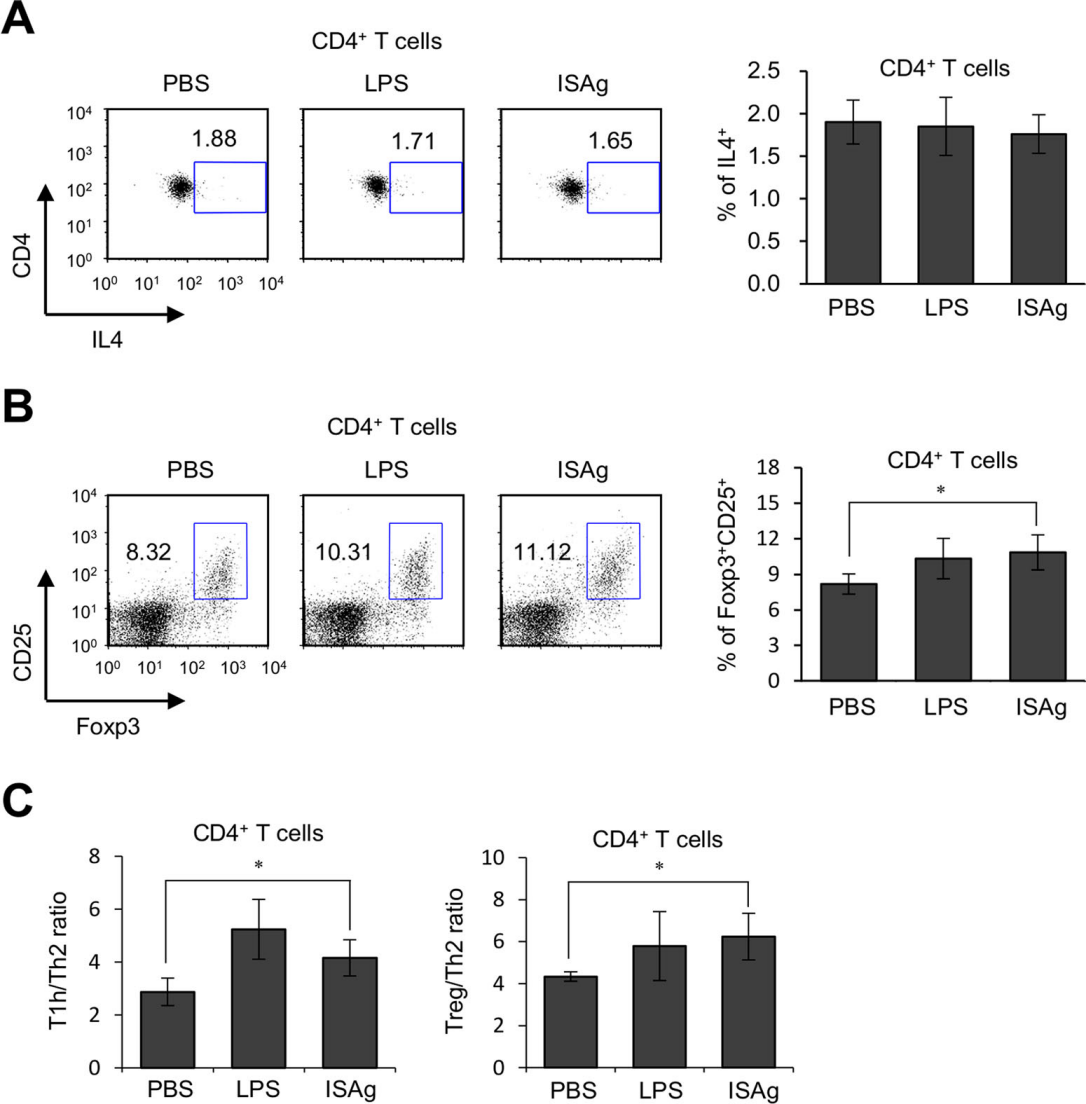
Oral administration of ISAg induces Treg expansion *in vivo*. (A-C) Yet40 B6 mice were given 4 mg ISAg or PBS by oral gavage three times per week for 4 weeks. Positive control mice were injected (i.p.) with 2 μg LPS once per week for 4 weeks. Four weeks later, the levels of IL4 (A) and Foxp3 (B) in CD4^+^ T cells were measured by flow cytometry. (C) The Th1/Th2 ratio was calculated as the percentage of IFNγ- and IL4-producing CD4^+^ T cells, and the Treg/Th2 ratio was calculated as the percentage of Foxp3- and IL4-producing CD4^+^ T cells. The results are presented as the mean ± standard deviation (*n* = 4 per group; Student's *t* test; *P < 0.05).

Next, we investigated whether ISAg could influence other CD4^+^ helper T cells such as Th2 and Tregs. ISAg was administered orally to Yet40 mice, and 4 weeks later, the levels of IL4 and Foxp3 in CD4^+^ T cells were measured by flow cytometry. ISAg treatment significantly increased Foxp3 levels, but not IL4 levels, in CD4^+^ T cells (Figure 5A and B). Moreover, the Th1/Th2 and Treg/Th2 ratios among CD4^+^ T cells increased in ISAg-treated mice compared to PBS-treated control mice (Figure 5C). Several studies have previously demonstrated that an increase in bifidobacteria and lactic acid bacteria can induce the expansion of the Treg cell population (Kwon et al. 2010; Shin et al. 2016). Thus, it will be of interest to further explore whether there are any changes in the composition of gut microbiota after the oral administration of ISAg. Moreover, because it has been reported that both Th1 and Treg cells inhibit the induction of Th2 responses in allergic diseases such as AD, these data suggest that ISAg may be useful for the treatment of allergic diseases.

## Supplementary Material

Supplemental Material

Supplemental Material

Supplemental Material
