# Supplementary figures and images for: TLR4-dependent effects of ISAg treatment on conventional T cell polarization in vivo
Source: Anim Cells Syst (Seoul). 2019 Apr 25;23(3):184–91. doi: 10.1080/19768354.2019.1610059 (PMC6566868; doi:10.1080/19768354.2019.1610059)

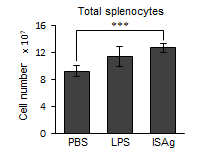

Supplement: Supplemental Material [file TACS_A_1610059_SM0176.tif]

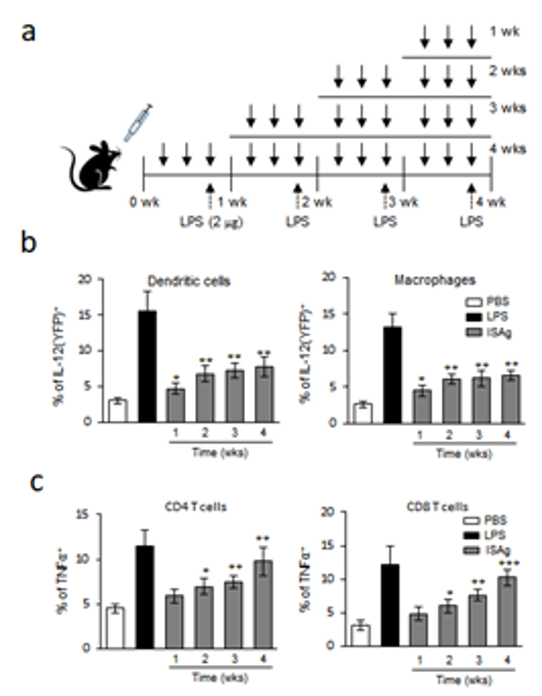

Supplement: Supplemental Material [file TACS_A_1610059_SM0174.tif]

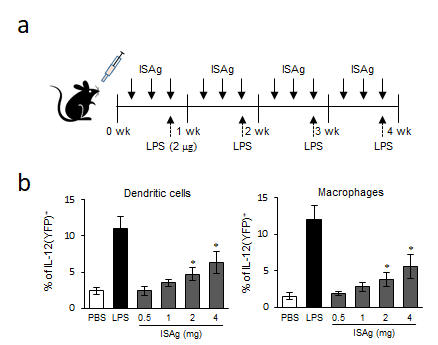

Supplement: Supplemental Material [file TACS_A_1610059_SM0173.tif]
